# Treatment of Multidrug-Resistant and Extensively Drug-Resistant Tuberculosis in Children: The Role of Bedaquiline and Delamanid

**DOI:** 10.3390/microorganisms9051074

**Published:** 2021-05-17

**Authors:** Francesco Pecora, Giulia Dal Canto, Piero Veronese, Susanna Esposito

**Affiliations:** Pediatric Clinic, Department of Medicine and Surgery, Pietro Barille Children’s Hospital, University Hospital of Parma, 43126 Parma, Italy; cescopec@hotmail.it (F.P.); giu.dalcanto@gmail.com (G.D.C.); piero.veronese@studenti.unipr.it (P.V.)

**Keywords:** multidrug-resistant tuberculosis, extensively drug-resistant tuberculosis, bedaquiline, delamanid, children

## Abstract

Multidrug-resistant (MDR) tuberculosis (TB) has been emerging at an alarming rate over the last few years. It has been estimated that about 3% of all pediatric TB is MDR, meaning about 30,000 cases each year. Although most children with MDR-TB can be successfully treated, up to five years ago effective treatment was associated with a high incidence of severe adverse effects and patients with extensively drug-resistant (XDR) TB had limited treatment options and no standard regimen. The main objective of this manuscript is to discuss our present knowledge of the management of MDR- and XDR-TB in children, focusing on the characteristics and available evidence on the use of two promising new drugs: bedaquiline and delamanid. PubMed was used to search for all of the studies published up to November 2020 using key words such as “bedaquiline” and “delamanid” and “children” and “multidrug-resistant tuberculosis” and “extensively drug-resistant tuberculosis”. The search was limited to articles published in English and providing evidence-based data. Although data on pediatric population are limited and more studies are needed to confirm the efficacy and safety of bedaquiline and delamanid, their use in children with MDR-TB/XDR-TB appears to have good tolerability and efficacy. However, more evidence on these new anti-TB drugs is needed to better guide their use in children in order to design effective shorter regimens and reduce adverse effects, drug interactions, and therapeutic failure.

## 1. Introduction

Tuberculosis (TB) causes even more deaths each year than any other bacterial infection [1]. In 2018, the World Health Organization (WHO) estimated 10.0 million new cases of TB (range 9–11.1 million). The burden of disease is very heterogeneous among countries, with the global average being around 130 new cases per 100,000 population per year. In the same period, TB deaths among HIV-negative people, in 2018, were estimated to be 1.2 million (range 1.1–1.3 million), and 251,000 deaths (range 223,000–281,000) among HIV-infected people. Children (aged <15 years) accounted for 11% of all TB cases in 2018, with higher rates in developing countries [1,2]. Indeed, in Africa, children contribute approximately 30% of incident TB cases. In countries with high HIV prevalence, the peak age prevalence of TB has shifted towards younger adults. These adults are often parents of young children, increasing the exposure of children to TB [3,4].

Multidrug-resistant (MDR) TB has been emerging at an alarming rate over the last few years. Annually, the new cases of MDR and rifampicin-resistant (RR) TB are estimated to be 500,000 [5]. Although an increased number of children is now being diagnosed and treated for TB, a low number is diagnosed for MDR-TB and little data are available on the occurrence of MDR-TB in children [6,7]. It has been estimated that about 3% of all pediatric TB is MDR, meaning about 30,000 cases each year [8]. MDR-TB is due to strains that are resistant to both isoniazid and rifampicin, while extensively drug-resistant (XDR) TB is characterized by MDR strains which present additional resistance also to fluoroquinolones and second level injectable drugs. Pre-XDR-TB denotes resistance to isoniazid and rifampicin, as well as to at least one fluoroquinolone or one second-line injectable drug.

The main obstacles facing treatment of MDR- and XDR-TB are the low availability of diagnostic tests and lengthy expensive treatment, with frequent appearances of adverse effects, drug interactions, and high rates of therapeutic failure. Although most children with MDR-TB can be successfully treated, up to five years ago effective treatment was associated with a high incidence of severe adverse effects and patients with XDR-TB had limited treatment options and no standard regimen [9,10,11,12].

In this context, over the last few years, the approval of new and repurposed drugs for the treatment of MDR-/XDR-TB have opened the possibility of new effective regimens. The main aim of this manuscript is to discuss our present knowledge of the management of MDR-TB disease in children, focusing on two promising new drugs: bedaquiline and delamanid. All the studies performed using bedaquiline and delamanid have been considered: this manuscript summarizes the main 12 published studies related to adults, as well asl the five studies already published and the five ongoing studies regarded the pediatric population.

## 2. Management of MDR-TB in Pediatrics

In most cases, a child develops MDR-TB following exposure to an infectious MDR-TB case (primary resistance). Furthermore, as a result of the poor adherence to the therapy or of inadequate or inconsistent therapy, a child with drug-susceptible TB can develop resistance to isoniazid and rifampicin (acquired resistance) [13,14].

Although the efficacy of preventive therapy for child contacts of drug-susceptible TB is well established, preventive treatment of children exposed to MDR-TB is considered on a case-by-case basis for evaluating the risk of infection and of disease progression. However, children in direct household contact with an adult with TB have a high risk of developing the disease and the treatment of MDR-TB in children is complex, long, and associated with frequent and significant side effects. For this reasons, prevention of MDR-TB in children is very important. In two ongoing clinical trials (TB-CHAMP (ISRCTN92TG634082) and PHOENIx MDR-TB (NCT03568383)) the administration of levofloxacin or delamanid, respectively, as MDR-TB preventive therapy in children is being evaluated [15,16].

Similar to adults, even in the pediatric population, the design of an appropriate MDR-TB treatment regimen should consider the following [17,18]: the likely or confirmed infecting *Mycobacterium tuberculosis* strain, through a drug susceptibility test (DST); previously failed TB treatment either in the child or in a known source case; the resistance profile in the relevant geographic area; the side and the severity of disease; the age of the child; and the ability to take certain drugs or drug formulation.

For MDR-TB, longer treatment regimens are recommended, and the choice of an effective all-oral regimen is suggested in several guidelines. The groupings of medicines recommended for use in longer MDR-TB regimens are reported in Table 1. To build an effective MDR-TB regimen for children, the WHO Groups A and B should be prioritized, as well as delamanid in children aged more than 3 years of age [19]. *The Sentinel Project for Pediatric Drug-Resistant Tuberculosis Field Guide* provides a suggestion on how to build therapeutic regimens in children based on the age and on the presence of fluoroquinolone resistance. Since TB in children is frequently non-severe and paucibacillary, the duration of treatment should depend upon the site of infection, the severity of disease, and the extent of drug resistance; in children with non-severe disease, treatment can last for 9 to 12 months, while in children with severe disease, a treatment of 12–18 months is required. Injectable-free regimens, especially in very young children and in those with mild disease, should be preferred in order to preserve hearing function. Therefore, greater knowledge of the efficacy and safety of the new drugs bedaquiline and delamanid and the possibility of their use in children may help to treat MDR-TB with all-oral shorter effective and safe regimens [20,21].

## 3. Bedaquiline

### 3.1. Mechanism of Action and Pharmacokinetics

**Figure 1:**
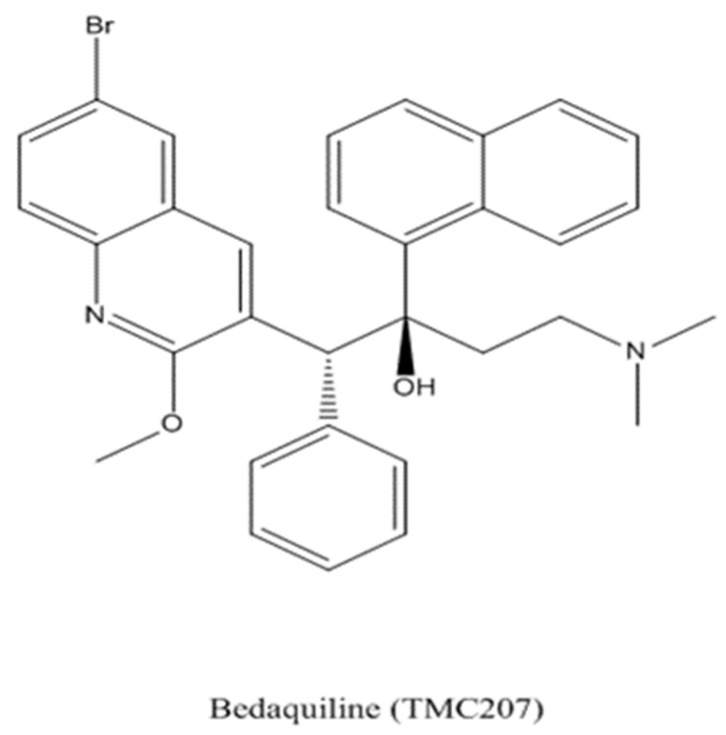
Bedaquiline structural formula.

Bedaquiline (molecular formula C_32_H_31_BrN_2_O_2_) is the first antitubercular agent approved by the FDA since the late 1970s [22]. Previously named TMC207 and R207910, it belongs to the family of diarylquinoline. It has a central nucleus of quinolone with lateral aminic and alcoholic chains. Figure 1 shows its structural formula.

Bedaquiline has a strong bactericidal activity as an inhibitor of mycobacterial ATP synthase proton pump [23,24,25]. In fact, it is the only anti-tubercular drug targeted to the energetic metabolism of mycobacteria. It also has an effect on further remodeling bacterial metabolism, which may be due to multiple factors, such as decreasing cellular ATP levels or changes in intracellular redox state [26]. Pharmacokinetic studies in humans have shown that bedaquiline is well absorbed after a single oral administration with a median time to reach peak concentration of 5–6 h after the dose and an effective half-life of more than 24 h [27,28]. The long terminal half-life in humans makes possible intermittent drug administration when combined with other drugs in the regimen against MDR-TB and XDR-TB [29].

### 3.2. Efficacy

There are numerous observational studies that have reported the compassionate use of bedaquiline culture conversion rates at Week 24 of treatment exceeding 90% [30,31,32,33]. Among these, the main ones are reported in Table 2.

It is important to report a retrospective study supported by *Médecins sans Frontières* in which MDR-TB treatment containing bedaquiline given through compassionate use programs [34] with linezolid and/or imipenem showed relatively good success rates in this cohort of previously treated patients with extensive and highly resistant TB (54/64–4.4% patients with a positive culture at treatment initiation culture converted; overall success rate 48/82–58.5%) [35]. This figure is confirmed by one of the most extensive retrospective studies conducted in 25 centers and 15 countries and including 428 patients with MDR-TB (45.6% XDR-TB and 22.1% HIV positive), in which success was achieved in 71.3% of patients treated with bedaquiline-containing regimes [36].

It is also interesting to note that, in these results, the highest success rates were achieved in patients with XDR-TB (76.9%) as compared with patients with MDR-TB (67.2%). Prospective clinical studies by Diacon et al. showed a faster crop conversion of the excretion as well as a higher conversion rate as compared with the placebo group [37,38,39]. In the multicenter phase 2 study of Pym et al. conducted on 233 patients (63.5% with MDR-TB, 18.9% with pre-XDR-TB, and 16.3% with XDR-TB), 87.1% of which had previously received second-line treatment, cultural conversion after 120 weeks of treatment occurred in 72.2% of patients treated; the highest success rate was observed in patients with MDR-TB (73.1%) and 23 of 38 patients with XDR-TB (61%) had a response at 120 weeks after the initiation of treatment [40].

### 3.3. Safety and Tolerability

Among the side effects associated with the use of bedaquiline, most effects reported in the literature are nausea, headache, and arthralgia. It is known that bedaquiline can interfere with cardiac electrical activity by lengthening the QT interval. Furthermore, it is good to remember that these drugs are used in combination with others potentially able to lengthen the QT range, such as clofazimine and fluoroquinolones [41,42]. Current EMA recommendations include the execution of an ECG track before starting treatment with bedaquiline and at least at Weeks 2 and 4, and every following month [43]. Weekly ECG monitoring can be considered if the patient presents other possible risk factors for QT interval lengthening, such as other medications, hypokalemia, hypomagnesemia, hypocalcemia, congenital syndrome of long QT, hypothyroidism, radiation, and heart failure. However, cardiac safety information regarding bedaquiline is limited, often because the monitoring activities have not been reported in a systematic way and in sufficient detail to be easily compared, while numerous studies do not report relevant information about patients’ QTc or heart safety more generally.

Bedaquiline remains a well-tolerated drug and a retrospective cohort study found that bedaquiline-containing regimens were associated with a large reduction in mortality in patients with drug-resistant tuberculosis as compared with regimens that did not contain bedaquiline [44]. In an individual patient data meta-analysis, of 12,030 patients treated for MDR-TB assessed from 25 countries in 50 studies, bedaquiline resulted in being one of the drugs analyzed associated with greater treatment success and reduced death [45]. Further studies have confirmed the safety of bedaquiline in varied settings; a recent prospective comparative study reporting long-term (24-month) treatment-related outcomes in patients with XDR-TB, treated with and without bedaquiline, showed that mortality in the bedaquiline group was more than halved [46,47]. Interruption of bedaquiline occurred in small percentages of patients, of which a minority lengthened the QT range. However, potential adverse effects are one of the main reasons that still limit its use in children [47].

### 3.4. Drug Interactions

Bedaquiline undergoes oxidative metabolism via the CYP3A4, with the formation of N-mono-desmethyl metabolite (M2), which has a high half-life (~230 h) but does not seem to contribute significantly to clinical efficacy. However, clearance of M2 is also thought to be mediated by CYP3A4 [44,48,49]. For this reason, as reported in the WHO interim policy guidance, caution should be exercised in case there is co-administration of bedaquiline and drugs that induce CYP3A (e.g., rifampicin), as it may decrease bedaquiline plasma concentrations resulting in reduced efficacy. Conversely, when bedaquiline is administered together with drugs that may inhibit liver function (e.g., ketoconazole or lopinavir/ritonavir), its plasma concentration may be increased, resulting in toxicity [50].

Limited data concerning the interactions between bedaquiline and antiretroviral medicines are available, and most of these are based on studies conducted in healthy volunteers [51,52,53]. In an observational study performed in HIV-infected and HIV-seronegative adult patients (age ≥18 years) with MDR-TB, Brill et al. showed that nevirapine did not show a relevant effect on bedaquiline and M2 exposure in HIV/MDR-TB co-infected patients. Conversely, ritonavir-boosted lopinavir (LPV/r) significantly increased bedaquiline concentration in HIV/MDR-TB co-infected patients, thus, an adjusted bedaquiline dosing regimen was suggested [54].

Therefore, according to the WHO interim policy guidance, patients with HIV who receive bedaquiline as part of MDR-TB treatment should have their antiretroviral (ART) regimens designed in close consultation with HIV clinicians and ART specialists.

### 3.5. Bedaquiline Treatment in the Pediatric Population

There are few data on the use of bedaquiline in pediatric population, as all patients <18 years were excluded in the first trials that evaluated and led to the approval of its use in MDR-/XDR-TB [34]. However, Aschar et al. reported on 27 cases in which children and adolescents with MDR-/XDR-TB have been treated with drug regimens that included bedaquiline and described good treatment responses and no cessation caused by adverse effects [55]. Furthermore, Conradie et al., in an open-label, single-group study in which patients ≥14 years of age were eligible for enrollment, showed that the combination of bedaquiline, pretomanid, and linezolid was effective in a high percentage of patients with XDR-TB. This regimen was associated with toxic effects (19 patients, 17%, had serious adverse events), with a high percentage related to linezolid and manageable. No patient had a significant increase in the QT interval related to bedaquiline [56]. Two ongoing pharmacokinetic trials [57,58], a Janssen-sponsored study and a study of the IMPAACT network, are testing optimal dosing and safety of bedaquiline among children and adolescents <18 years of age. According to interim results from these studies, in the 2020 WHO consolidated guidelines on tuberculosis, optimal bedaquiline doses are reported that can be used safely in children aged >5 years [59]. Table 3 and Table 4 summarize main data on bedaquiline obtained in children and adolescents, whereas Table 5 shows the recommended dosing.

## 4. Delamanid

### 4.1. Mechanism of Action and Pharmacokinetics

**Figure 2:**
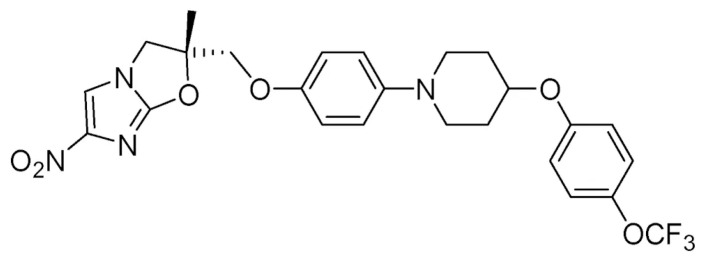
Structural formula of delamanid.

Delamanid (molecular formula C_25_H_25_F_3_N_4_O_6_) is a nitroimidazole agent that inhibits synthesis of mycolic acids (specifically methoxy-mycolic acid and keto-mycolic acid), leading to depletion of mycobacterial cell wall components and destruction of the mycobacteria, with bactericidal activity [60,61]. It is thought to be a prodrug that requires biotransformation via the mycobacterial F420 coenzyme system to mediate its antimycobacterial activity against mycobacteria [62]. Mutations in one of coenzyme F420 genes has been proposed as the mechanism of resistance to delamanid [62] and the spontaneous rate of resistance has been reported as from 6.44 × 10^−6^ to 4.19 × 10^−5^ [63]. Figure 2 shows its structural formula.

In humans, delamanid absorption was almost two-fold higher when administrated with meals as opposed to in a fasted state [64]. The pharmacokinetics of delamanid are nonlinear, i.e., doubling the dose results in less than twice the exposure. Plasma concentration peaks at around 4–8 h after oral dosing with a half-life of 30–38 h [64]. The absolute oral bioavailability in humans is estimated to range from 25 to 47% [63]. Delamanid has a large apparent volume of distribution (Vz/F of 2100 L) with a binding to total proteins of ≥99.5% [65]. It is primarily metabolized in plasma by albumin, a non-hepatic process, rather than cytochrome P450 enzymes [65]. It is excreted primarily in the stool, with approximately 6% excretion in the urine [66]. Animal studies have indicated that delamanid can pass brain and placental blood barriers and is also excreted in breast milk [66].

### 4.2. Efficacy

Preclinical studies have shown in vitro activity against MDR-TB of delamanid [67,68]. In mouse and guinea pig models, adding delamanid to the MDR-TB treatment regimen resulted in significantly accelerated eradication of bacilli [69]. On the basis of the promising preclinical data, delamanid was moved into clinical development (Table 6). A trial to assess early bactericidal activity (EBA) in a group of uncomplicated, smear-positive, pulmonary TB adult patients showed a steady decline in CFU from baseline over 14 days of daily delamanid monotherapy [67].

A phase IIb, randomized, placebo-controlled, multinational clinical trial, showed that delamanid increased the two-month sputum culture conversion rates when added to an optimized background regimen (OBR) in adult MDR-TB patients [70]. Among patients who received a background drug regimen plus delamanid, 45.4% had sputum-culture conversion at 2 months as compared with 29.6% of patients who received a background drug regimen plus placebo. Other phase IIb trials comparing patients receiving 6 months (i.e., 6 or 8 months) of treatment with delamanid in combination with OBR to patients receiving 2 months of treatment (i.e., 0 or 2 months) showed that the first group had a significantly higher proportion of favorable outcomes (75% vs. 55%) and lower mortality (3% vs. 12%) [71,72]. In contrast, a recent large randomized, double-blind, placebo-controlled trial showed that the reduction in median time to sputum culture conversion over 6 months was not significant in the delamanid arm. It is important to note that this result could be misrepresented by the over performance of the placebo, given the already highly effective background treatment [73].

### 4.3. Safety and Tolerability

Preclinical studies have shown that delamanid is well tolerated, and likely has no genotoxicity or carcinogenicity [64]. During the first trial in humans, in which delamanid was administrated in daily monotherapy for 14 days to MDR-TB adult patients, no serious treatment-emergent adverse events (AEs) occurred [67]. In a three-month randomized, double-blind, placebo-controlled, phase IIb trial on MDR-TB adult patients, treatment emergent AEs for which the incidence in the delamanid arm was higher by >5% as compared with the placebo included: nausea, vomiting, headache, and prolonged QTcF interval [70]. No clinical cardiac events were reported. Data on safety of delamanid in children are still forthcoming as several clinical trials are in the process of recruiting patients and have not yet published their results (Table 8).

Few pediatric case reports or case series are reported in the literature. In a study that enrolled 16 children treated with delamanid on compassionate basis, no adverse event was reported in fifteen of them, while a child treated with a combination of delamanid, capreomycin, ethionamide, cycloserine, clofazimine, imipenem, amoxicillin/clavulanate, and pyrazinamide experienced vomiting, renal impairment, electrolyte disturbances, and prolonged QTc on the Fridericia formula [74]. In another case series, 36 children (0–17 years) treated with delamanid at two sites in the Philippines and one site in South Africa well tolerated the treatment [75]. Shah et al. described two children with XDR-TB treated with bedaquiline and delamanid who developed prolonged QTc on the Bazett formula but normal QTc on the Fridericia formula, without any other adverse effects [76].

Since the primary safety concern of delamanid is QTc prolongation, ECG and electrolytes should be monitored before starting treatment. As hypoalbuminemia may increase the potential of delamanid to prolong QT, serum albumin levels should also be monitored [62]. Although the reported cases do not show a high incidence of adverse effects, the limited data available does not allow definite conclusions to be drawn on the safety of the drug. Thus, close monitoring and further investigations are needed to establish the safety profile in the pediatric population.

### 4.4. Drug Interactions

As reported above, delamanid is largely metabolized by albumin rather than cytochrome P450 enzymes, making it an attractive option to minimize drug–drug interactions [61]. There is no known drug–drug interaction with antiretroviral drugs [77].

Caution should be applied while combining delamanid with other drugs that have the potential of prolonging QT. The recent data review for the WHO guidelines suggested that there are no additional safety concerns for concurrent use of delamanid with bedaquiline [19]. The combined QT effects of bedaquiline and delamanid, compared with bedaquiline or delamanid alone (added to multidrug background therapy), were evaluated in a randomized controlled trial of 75 patients (>3000 ECGs). The average QTcF prolongation attributable to bedaquiline was 12.3 ms, and to the combination of bedaquiline and delamanid was 20.7 ms. No participants had grade 3 or 4 of QT prolongation [78].

### 4.5. Delamanid Treatment in the Pediatric Population

The first case of a child treated with delamanid documented a favorable clinical, microbiological, and radiological response [79,80]. A subsequent case series, describing 16 children who received delamanid on a compassionate basis, reported a favorable outcome in terms of culture conversion in all of them [74]. Another study reported favorable outcomes in two children with PreXDR-TB and XDR-TB after 24 weeks of treatment with delamanid plus background regimen [81]. Furthermore, results of other retrospective studies in which pediatric patients were included in delamanid-treated cohorts, have shown good tolerability and treatment response [82,83].

In 2016, the WHO issued an interim policy guidance on the use of delamanid in the treatment of MDR-TB in children and adolescents [84], recommending that delamanid may be added to background regimen for MDR-/XDR-TB for those patients who have previously received treatment with second-line anti-TB medicines, or who have isolates with additional resistance to fluoroquinolones or second-line injectable agents, or in whom components of the shorter MDR-TB regimen were contraindicated. Children with QTcF > 500 msec should not receive the drugs; moreover, proper pharmacovigilance and monitoring of adherence should be ensured [84].

Although results are encouraging, data on delamanid efficacy in children are still scarce and further research is needed. As such, current WHO guidelines recognize delamanid as a Group C drug in children aged 3 years or more [19]. Table 7 summarizes main data on delamanid obtained in children and adolescents, More evidence on the efficacy of delamanid would be helpful to better guide its use and several clinical trials with this purpose are ongoing [85,86,87] (Table 8). Table 9 summarizes recommended dosing. Administration in children <6 years may be difficult because 50 mg tablets cannot be split accurately (may affect bioavailability); in addiction, the contents are bitter and unpalatable. Treatment should continue for 6 months (intensive phase) and be given after meals.

## 5. Conclusions

TB is still one of the most difficult infectious diseases to treat, and the second most frequent cause of death due to infectious disease throughout the world. The number of cases of MDR-/XDR-TB, which are characterized by high mortality rates, is increasing. The therapeutic management of children with MDR- and XDR-TB is complicated by a lack of knowledge, and the fact that many potentially useful drugs are not registered for pediatric use and there are no formulations suitable for children in the first years of life. Furthermore, most of the available drugs are burdened by major adverse events that need to be taken into account, particularly in the case of prolonged therapy. Interestingly, studies around the world have shown controversies in the definition of bedaquiline and delamanid resistance [88,89]. Unfortunately, up to now, resistance to both of the drugs has been reported in vitro, but standardized drug susceptibility testing has not been developed and agreed upon. Known mechanisms of resistance to bedaquiline include mutations within the *atpE*, *Rv0678*, and *pepQ* genes [88], whereas delamanid-resistant bacilli have mutations in one of the five genes in the F420-dependent bio-activation pathway [89].

Although data on pediatric population are limited and more studies are needed, our review shows the efficacy and safety of bedaquiline and delamanid in adolescent children with MDR-TB and XDR-TB. However, more evidence on these new anti-TB drugs is needed to better guide their use in children, design effective shorter regimens and reduce adverse effects, drug interactions, and therapeutic failure. Furthermore, the development of standardized drug susceptibility testing for bedaquiline and delamanid is urgently needed.

## Figures and Tables

**Table 1:** The World Health Organization (WHO) groups of multidrug-resistant TB drug.

Groups and Steps	Medicine	Abbreviation
**Group A:** **Include all three medicines**	Levofloxacin or moxifloxacin	Lfx Mfx
	Bedaquiline	Bdq
	Linezolid	Lzd
**Group B:** **Add one or both medicines**	Clofazimine	Cfz
	Cycloserine or terizidone	Cs Trd
**Group C:** **Add to complete the regimen, and when medicines from Groups A and B cannot be used**	Ethambutol	E
	Delamanid	Dlm
	Pyrazinamide	Z
	Imipenem-cilastatin or meropenem	Ipm-Cln Mpm
	Amikacin (or streptomycin)	Am (S)
	Ethionamide or prothionamide	Eto Pto
	P-aminosalicylic acidi	PAS

**Table 2:** Principal studies on the effectiveness of bedaquiline treatment in adults.

Authors [Reference] (Year)	Type of Study	Study Population (Age of Patients)	Therapy	Results
Diacon et al. [37] (2009)	phase 2, randomized, double-blind, controlled trial	47 patients with MDR pulmonary tuberculosis (18–65 years)	Bedaquiline (400 mg once daily for 2 weeks, followed by 200 mg three times a week for 6 weeks), or placebo, plus background regimen (8 weeks)	Reduction of time to induce sputum conversion, as compared with placebo (*p* = 0.003) Increased proportion of patients with conversion of sputum culture (48% vs. 9%)
Diacon et al. [39] (2014)	phase 2, randomized, double-blind, controlled trial	160 patients with MDR pulmonary tuberculosis (18–65 years)	Bedaquiline (400 mg once daily for 2 weeks, followed by 200 mg three times a week for 6 weeks), or placebo, plus background regimen (24 weeks)	Reduction of the median time to culture conversion, as compared with placebo, from 125 days to 83 days (*p* < 0.001) Increased rate of culture conversion at 24 weeks (*p* = 0.008) and at 120 weeks (*p* = 0.04).
Guglielmetti et al. [30] (2015)	retrospective cohort study	35 patients with MDR-TB (18–70 years)	Bedaquiline (400 mg once daily for 2 weeks, followed by 200 mg three times a week), plus background regimen	Culture conversion achieved in 28 of 29 (97%) cases with culture-positive pulmonary tuberculosis at bedaquiline initiation
Pym et al. [40] (2016)	phase 2, multicenter, open-label, single-arm trial	205 patients with MDR-TB (18–68 years)	Bedaquiline (400 mg once daily for 2 weeks, followed by 200 mg three times a week for a further 22 weeks), plus background regimen	Culture conversion was 72.2% at 120 weeks: MDR-TB (73.1%), pre-XDR-TB (70.5%) XDR-TB (62.2%)
Guglielmetti et al. [31] (2017)	multicentre observational study	45 patients with MDR-TB (30–42 years)	Bedaquiline (400 mg once daily for 2 weeks, followed by 200 mg three times a week) -12 patients: standard bedaquiline treatment (≤ 190 days) -33 patients: prolonged bedaquiline treatment (>190 days)	36 patients (80%) had favourable outcome 5 were lost to follow-up 3 died 1 failed and acquired bedaquiline resistance
Olaru et al. [32] (2017)	retrospective cohort study	30 patients with MDR/XDR-TB (23–39 years)	6 months of a bedaquiline-containing regimen	Culture conversion achieved within 8 weeks of initiating MDR-TB treatment in 12 (60%) patients and within 6 months in 20 (100%) patients
Borisov et al. [36] (2017)	large, retrospective, multicenter observational study	428 patients with MDR/XDR-TB (27–44 years)	Bedaquiline (400 mg once daily for 2 weeks, followed by 200 mg three times a week), plus background regimen	Culture conversion rates: 30.1% at 30 days 56.7%, at 60 days 80.5%, at 90 days 91.2% at the end of treatment
Hewison et al. [35] (2018)	retrospective cohort study	82 patients with MDR-TB/pre-XDR-TB/XDR-TB (31–51 years)	Bedaquiline (400 mg once daily for 2 weeks, followed by 200 mg three times a week)	Culture conversion achieved in 54/64 (84.4%) patients with a positive culture at treatment initiation

MDR, multidrug-resistant; TB, tuberculosis; XDR, extensively drug-resistant.

**Table 3:** Principal studies on bedaquiline in children and adolescents (0–18 years old) with MDR-TB.

Authors (Year)	Type of Study	Study Population	Median Age of Patients (Range)	Therapy	Results
Aschar et al. [55] (2017)	retrospective cohort study	27 patients with confirmed or presumed MDR/XDR-TB	16 years (10–17 years)	Bedaquiline (400 mg once daily for 2 weeks, followed by 200 mg three times a week for 24 weeks), plus background regimen One 10-years-old girl (weighing 35 kg) received 300 mg daily during her loading phase	Sputum culture negative: 23/23 (100%) and No clinical signs suggestive of treatment failure 5 patients (19%) reported adverse effects caused by bedaquiline (prolongation of QTc), without correlated symptoms
Conradie et al. [56] (2020)	Open-label, single-group study	109 patients with MDR/XDR-TB	35 years (17–60 years)	Bedaquiline (400 mg once daily for 2 weeks, followed by 200 mg three times a week for 24 weeks) + Pretomanid (200 mg daily for 26 weeks) + Linezolid (1200 mg daily for 26 weeks)	Unfavourable outcome: 11 patients (10%) vs. Favourable outcome: 98 patients (90%) Serious adverse events: 19 patients (17%) No patient had a QT interval increase > 480 msec.

MDR, multidrug-resistant; TB, tuberculosis; XDR, extensively drug-resistant.

**Table 4:** Ongoing trials testing bedaquiline pharmakokinetic in children and adolescents (0–18 years old) with MDR-TB.

Title	Study Design	Target Population	Intervention	Outcome Measures
Janssen C221 (NCT02354014)	A phase II, open-label, multicenter, single-arm study	Children and adolescents who have confirmed or probable pulmonary MDR-TB enrolled in 4 age-based cohorts: (1) ≥12–<18 years (2) ≥5–<12 years (3) ≥2–<5 years (4) 0 months–<2 years	Cohort 1: bedaquiline 400 mg qd, for first 2 weeks, followed by 200 mg tiw for 22 weeks Cohort 2: bedaquiline 200 mg qd for first 2 weeks, followed by 100 mg tiw for 22 weeks. Cohort 3: bedaquiline 8 mg/kg qd for the first 2 weeks, followed by 4 mg/kg tiw for 22 weeks. Cohort 4: bedaquiline dose will be selected based on the results from the previous cohorts 1, 2 and 3. + BR MDR-TB Medications.	Pharmacokinetic, safety, tolerability, and antimycobacterial activity of bedaquiline
IMPAACT P1108 (NCT02906007)	Phase I/II, open-label, single-arm study to evaluate	HIV-infected and HIV-uninfected infants, children, and adolescents with MDR-TB disease. *Age at enrollment*: Cohort 1: ≥6–<18 years Cohort 1: ≥2–<6 years Cohort 1: 0–<2 years *Weight at enrollment:* Cohort 1: ≥15 Cohort 1: ≥7 Cohort 1: ≥3	Bedaquiline doses will vary based on the participant’s age and weight. + optimized individualized MDR-TB therapy	Pharmacokinetic, safety, and tolerability of bedaquiline

BR, background regimen; qd, once daily; tiw, 3 times per week; MDR, multidrug-resistant; TB, tuberculosis.

**Table 5:** Bedaquiline recommended dosing.

Bedaquiline	≥15 Years	< 15 Years	
		16–30 kg	>30 kg
**100 mg tab**	4 tabs qd for first 2 weeks; then 2 tabs qd M/W/F for 22 weeks	2 tabs qd for 2 weeks; then 1 tab qd M/W/F for 22 weeks	4 tabs qd for 2 weeks; then 2 tabs qd M/W/F for 22 weeks
**20 mg dt**		10 dts qd for 2 weeks; then 5 dts od M/W/F for 22 weeks	20 dts qd for 2 weeks; then 10 dts od M/W/F for 22 weeks

Dt, dispersible tablet; qd, once daily; M/W/F, Monday, Wednesday, Friday; tab, tablet.4.

**Table 6:** Principal studies on the effectiveness of delamanid treatment in adults.

Authors [Reference] (Year)	Type of Study	Study Population (Age of Patients)	Therapy	Results
Diacon et al. [67] (2011)	phase IIa, open-label, randomised, controlled trial	48 patients with newly diagnosed smear-positive pulmonary TB (18–64 years)	four groups of 12 patients receiving (1) delamanid 100 mg qd (2) delamanid 200 mg qd (3) delamanid 300 mg qd (4) delamanid 400 mg qd + one control group of 6 patients receiving standard four-drug anti-tuberculosis treatment (HRZE)	Delamanid at all dosages demonstrated significant exposure dependent EBA over 14 days
Gler et al. [70] (2012)	Double-blind, multicenter, randomized, placebo-controlled trial	481 patients with pulmonary MDR-TB (18–64 years)	Group 1: Delamanid 100 mg td + BR Group 2: Delamanid 200 mg td + BR Group 3: Placebo + BR	Statistically significant difference in sputum-culture conversion between delamanid groups and placebo group
Skripconoka et al. [71] (2013)	Multicenter observational study	421 patients with pulmonary MDR-TB (18–63 years)	Group 1: Delamanid (100 mg td or 200 mg td) for ≥ 6 months + BR Group 2: Delamanid (100 mg td or 200 mg td) for < 6 months + BR	Favourable outcomes observed in patients who received delamanid for ≥6 months, compared to patients who received delamanid for ≤2 months. Reduction of mortality of 1.0% among those receiving long-term delamanid vs. short-term/no delamanid (*p* < 0.001)
Von Groote-Bidlingmaier et al. [73] (2019)	Phase III, randomised, double-blind, placebo-controlled trial	511 patients with a diagnosis of pulmonary MDR tuberculosis (18–69 years)	Group 1: Delamanid (100 mg td for 2 months followed by 200 mg qd for 4 months) + BR Group 2: Placebo + BR	No significant advantage in the delamanid arm in reducing the median time to sputum culture conversion

BR, background regimen; EBA, early bactericidal activity; HRZE, isoniazid/rifampicin/pyrazinamide/ethambutol; MDR, multidrug-resistant; qd: once daily; TB, tuberculosis; td, twice daily.

**Table 7:** Principal studies on delamanid in children and adolescents (0–18 years) with MDR-TB.

Authors (Year)	Type of Study	Study Population	Median Age of Patients (Range)	Therapy	Results
Esposito S et al. [79] (2016)	Case report	1 patient with confirmed pulmonary XDR-TB	12 years	Delamanid 100 mg td for 24 months + BR	Gastric aspirate culture negative after 1 week, the patient was considered cured at the end of the treatment. No adverse events were reported, normal corrected QT interval.
Tadolini M et al. [74] (2016)	Case series	16 patients with confirmed pulmonary MDR/XDR-TB (2 also had extrapulmonary TB)	15 years (8–14 years)	Delamanid 100 mg td for 24 weeks + BR (except one who received 50 mg td)	81.2% culture-negative; no or mild adverse events except one patient who experienced severe vomiting, renal impairment, hypokalaemia, hypomagnesaemia and QT interval prolongation
Kuksa L et al. [81] (2017)	Case series	2 patients with PreXDR/XDR-TB	12 years (11–13 years)	Delamanid for 24 weeks (dosage not reported) + BR	Both patients were considered cured at the end of the treatment. No adverse events were reported, normal corrected QT interval.

BR, background regimen; MDR, multidrug-resistant; qd, once daily; TB, tuberculosis; td, twice daily; XDR, extensively drug-resistant.

**Table 8:** Ongoing trials testing delamanid pharmakokinetic in children (0–18 years) with MDR-TB.

Title	Study Design	Target Population	Intervention	Outcome Measures
A 6-Month Safety, Efficacy, and pharmacokinetic Trial of Delamanid in Pediatric Patients With MDR-TB (NCT01859923)	Phase 2, Open-label, Multiple-dose Trial	Children and adolescents who have confirmed or probable pulmonary MDR-TB enrolled in 4 age-based cohorts: (1) 12 to 17 years (2) 6 to 11 years (3) 3 to 5 years (4) 0 to 2 years	Group 1: Delamanid 100 mg td for 182 days + BR for 365 days Group 2: Delamanid 50 mg td for 182 days + BR for 365 days Group 3: Delamanid 25 mg (pediatric formulation) td for 182 days + BR for 365 days Cohort 4: Delamanid from 5 mg qd to 10 mg td (pediatric formulation) based on weight measurements for 182 days + BR for 365 days	Pharmacokinetic, safety, tolerability, and efficacy of delamanid
Pharmacokinetic and Safety Trial to Determine the Appropriate Dose for Pediatric Patients with MDR-TB (NCT01856634)	Phase 1, Open-label, Multiple-dose, and Age De-escalation Trial	Children and adolescents who have confirmed or probable pulmonary MDR-TB enrolled in 4 age-based cohorts: (1) 12 to 17 years (2) 6 to 11 years (3) 3 to 5 years (4) 0 to 2 years	Group 1: Delamanid 100 mg td + BR for 10 days Group 2: Delamanid 50 mg td + BR for 10 days Group 3: Delamanid 25 mg (pediatric formulation) td + BR for 10 days Cohort 4: Delamanid from 5 mg to 10 mg td (pediatric formulation) based on weight measurements + BR for 10 days	Pharmacokinetics, Safety and Tolerability of Delamanid
Evaluating the Pharmacokinetics, Safety, and Tolerability of Delamanid in Combination With Optimized Multidrug Background Regimen for MDR-TB in HIV-Infected and HIV-Uninfected Children With MDR-TB (NCT03141060)	Phase I/II Open-Label, Single-Arm Study	Children and adolescents who have MDR-TB with and without HIV-infection, enrolled in 4 age-based cohorts: (1) 12 to 17 years (2) 6 to 11 years (3) 3 to 5 years (4) 0 to 2 years	Delamanid for 24 weeks + BR, dose based on age group and weight	Pharmacokinetics, Safety, and Tolerability of Delamanid

BR, background regimen; MDR, multidrug-resistant; qd:, once daily; TB, tuberculosis.

**Table 9:** Delamanid recommended dosing.

Delamanid		
Age (Weight Band)	Dose	50 mg Tablet
3–5 yrs (<24 kg)	25 mg twice daily	- ^a^
6–11 yrs (24–34 kg)	50 mg twice daily	1 tablet twice daily
12–17 yrs (>35 kg)	100 mg twice daily	2 tablets twice daily

^a^ Giving half a 50 mg adult tablet in these children does not result in the same blood levels observed in trials using the special 25 mg paediatric tablet. Bioavailability may further be altered when the 50 mg tablet is split, crushed or dissolved.

## Data Availability

All the available data have been included in this manuscript.
